# Bronchoscopy-guided bronchial epithelium sampling as a tool for selecting the optimal biologic treatment in a patient with severe asthma: a case report

**DOI:** 10.1186/s13223-019-0378-6

**Published:** 2019-11-27

**Authors:** Chin-Wei Kuo, Xin-Min Liao, Yi-Ching Huang, Han-Yu Chang, Chi-Chang Shieh

**Affiliations:** 1 Division of Chest Medicine, Department of Internal Medicine, National Cheng Kung University Hospital, Tainan, Taiwan; 2 Institute of Clinical Medicine, College of Medicine, National Cheng Kung University, 138 Sheng-Li Road, Tainan, 70403 Taiwan; 3 Department of Internal Medicine, National Cheng Kung University Hospital, Tainan, Taiwan; 4 Department of Pediatrics, National Cheng Kung University Hospital, Tainan, Taiwan

**Keywords:** Severe asthma, Omalizumab, Bronchial epithelium sampling

## Abstract

**Background:**

There are numerous biologics for treating patients with severe asthma. A cost-effective method for selecting the most appropriate biologic therapy for a patient is thus important. Bronchoscopy-guided bronchial epithelium sampling may provide information for determining the type of inflammation in the airways of severe asthma patients through immunochemical analysis and thus help clinicians select the correct biologics.

**Case presentation:**

We report the case of a female with severe asthma and eosinophilia who initially responded to omalizumab treatment. She developed an allergic reaction after four injections of omalizumab. Omalizumab desensitization was successfully conducted. To select an appropriate biologic agent after this hypersensitivity episode, we performed bronchoscopy-guided bronchial epithelium sampling. Omalizumab treatment was resumed based on the findings of immunohistochemical staining after a successful desensitization procedure, leading to long-term control of her severe asthma.

**Conclusions:**

Selecting an adequate biologic agent for severe, uncontrolled asthma is a challenge in clinical medical practice. Although phenotypes, blood eosinophils, and serum IgE levels have been proposed for use as a reference, there is a dissociation between the blood immune-cell level and the airway epithelium immune reaction, as confirmed in previous studies. Airway epithelium immunohistochemistry staining for targeted immune cells has been used to determine various types of airway inflammation; however, this technique is rarely used in a clinical setting. Previous studies have revealed the relative safety of performing bronchoscopy biopsies for patients with severe asthma. Among the sampling techniques used for tissue diagnosis, including nasal biopsies, nasal or bronchial brushing, and bronchoalveolar lavage, bronchoscopy-guided bronchial epithelium sampling provides more accurate information about the epithelial and inflammatory cells in the tissue context. It is thus a powerful tool for selecting the most suitable biologics in difficult clinical conditions.

## Background

The prevalence of asthma, a chronic inflammatory disease of the airways, has been increasing worldwide [1]. Severe asthma refers to asthma with uncontrolled symptoms despite treatment with medium- to high-dose inhaled corticosteroid (ICS) and long-acting beta-agonists (LABAs). An increasing number of biologic agents, such as anti-IgE and anti-IL-5 monoclonal antibody, have become clinically available. The selection of biologic agents has become an important issue for patients with severe asthma in terms of disease control and economic concerns. Herein, we report a case of severe asthma involving a clinical dilemma concerning the choice between a potentially allergenic biologic agent, anti-IgE, and an unproven one, anti-IL-5 agent. To resolve this dilemma, bronchoscopy-guided bronchial epithelium sampling was performed to determine the optimal biologic therapy.

## Case presentation

The patient was a 65-year-old housewife, who had never smoked, with a medical history of gastroesophageal reflux disease, allergic rhinitis, and asthma since childhood. The daily activities of the woman were limited due to her dyspnea on exertion, which was refractory to the treatment of high-dose ICS, LABA, and long-acting muscarinic-antagonist (LAMA). She needed frequent short-term oral corticosteroid (OCS) treatment for symptom control of her asthma, yet frequent exacerbations of her asthma remained. Furthermore, she required mechanical ventilation support 6 times in the past 3 years due to hypercapnic respiratory failure.

She was referred to our hospital in March 2017. Oral montelukast, theophylline, and famotidine were added to her original inhalation therapy to control her asthma. Repeated pulmonary function tests showed obstructive ventilatory deficit with positive bronchodilator response in terms of volume responder criteria (Additional file 1: Table S1). Laboratory profiles revealed eosinophilia, a mildly elevated serum IgE level (Additional file 1: Table S2), and negative multiple-antigen simultaneous test results. Due to the uncontrolled status of her severe asthma during follow-up, we initiated a biologic agent, namely omalizumab, with a monthly dose of 150 mg via subcutaneous injection 3 months after the referral. Symptoms of her asthma improved significantly after the second omalizumab dose. However, pruritic small erythematous papules developed over her trunk and extremities 2 weeks after the fourth injected dose of omalizumab (Additional file 2: Figure S1). Drug eruption was suspected based on the high Naranjo score. The patient underwent a right-thigh skin biopsy by dermatologist, the results of which were consistent with maculopapular drug eruption (Additional file 2: Figure S2). Omalizumab was hence discontinued under the suspicion of an omalizumab allergy.

After discussion with the patient, we performed omalizumab desensitization on September 19 and October 3, 2017, according to a previously reported protocol [2]. The process of desensitization proceeded smoothly without hypersensitivity responses. The patient remained stable during the following few months, and the skin rash disappeared 1 month after last dose of omalizumab exposure.

**Fig. 1:**
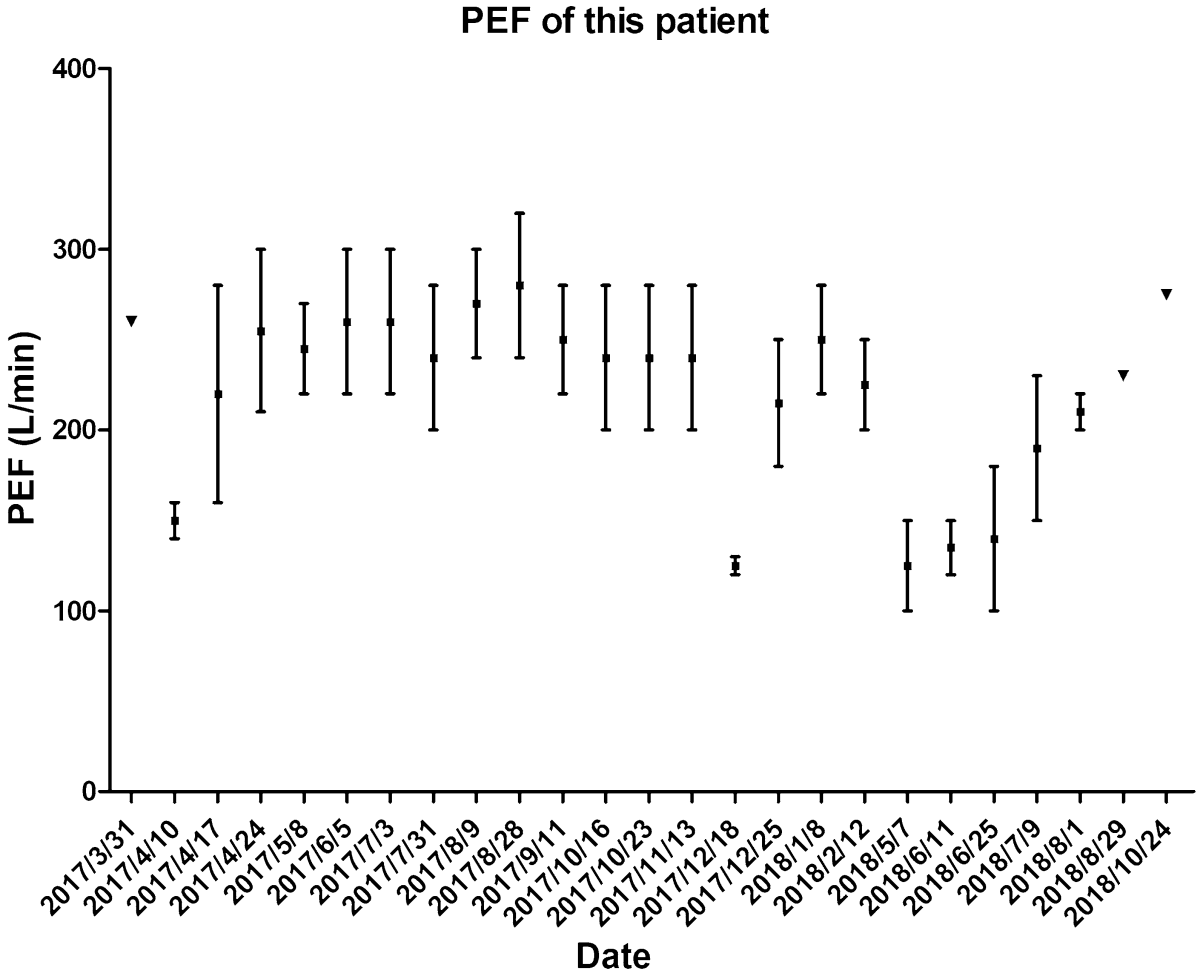
PEF level of the patient. The upper and lower bounds of the line represent the range of PEF change during the OPD follow-up period, and the square mark in the middle of the line represents the average maximum and minimum values of PEF during the period. Triangle marks represent the maximum PEF during the period on 2017/03/31, 2018/08/29, 2018/10/24. 2017/05/–2017/08 omalizumab use; 2017/09–2017/10 omalizumab desensitization; 2017/12/18 upper airway infection; 2018/05/07 LLL pneumonia; 2018/07/24 resume omalizumab use. *PEF* peak expiratory flow

**Fig. 2:**
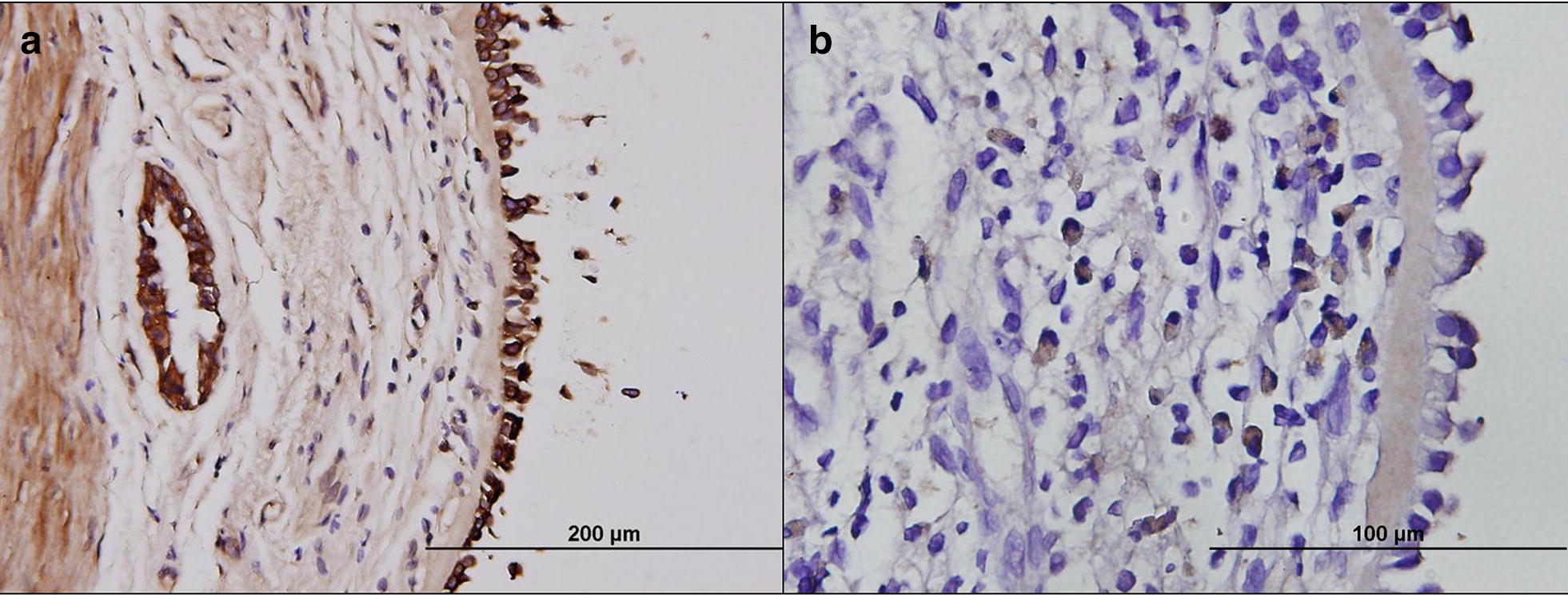
Immunohistochemistry staining of bronchial epithelium for the patient. **a** Significant IgE-positive epithelium cells in the specimen, indicating a strong IgE-mediate immune reaction in the patient’s airway. **b** Weak positive staining of IL-5 in the submucosal area

In May 2018, the patient suffered from fever and progressive dyspnea, during which time *Moraxella* (formerly *Branhamella*) *catarrhalis* pneumonia with lower-left-lung atelectasis was diagnosed using sputum microbiology and chest computed tomography. Her peak expiratory flow (PEF) value declined to around 100 to 150 L per minute (Fig. 1), and she became OCS-dependent for symptom control even after the pneumonia was resolved. The follow-up hemograms showed elevated eosinophil counts. To determine whether to resume the anti-IgE treatment or switch to anti-IL-5 monoclonal-antibody, we decided to perform bronchoscopy-guided bronchial epithelium sampling to identify the local airway inflammation according to a previously published protocol [3]. The immunohistochemical staining (Fig. 2) showed strongly positive staining of IgE over airway epithelium cells and only weakly positive immunohistochemical staining of IL-5 over the sub-mucosal area. According to the airway epithelial biopsy findings, we re-challenged omalizumab treatment in doses of 300 mg according to the patient’s serum IgE level and body weight in August 2018. The PEF and asthma symptoms improved after omalizumab was resumed for 2 months. The patient’s asthma has since remained under control with the treatment, including omalizumab.

## Discussion and conclusion

Severe asthma is a heterogeneous disease that is difficult to control despite medium- to high-dose ICS therapy. Poor control of asthma leads to high mortality and impaired quality of life, and increases personal and public health expenditure [1]. With the availability of biologics that target specific inflammatory mechanisms, it is important to select the right biologic for each severe asthma patient since they may have a distinct inflammatory mechanism underlying the common asthmatic symptoms [4]. Considering the poor correlation between blood eosinophilia and tissue eosinophilia, a previous study has shown that the blood eosinophil count is not predictive of the therapeutic response of the anti-IL-5 biologic agent in treating severe asthma [5]. This indicates that eosinophil is not the only effector cell in the inflammatory process of severe asthma [6]. An algorithm has been proposed for selecting the most suitable biologic agent for treating severe asthma based on the patient’s serum IgE level, blood eosinophil, and allergy history [7]. However, a dilemma may emerge for patients presenting with both allergic asthma and high blood eosinophil. Moreover, drug allergies complicate selection. Sequential trials of different biologic agents for asthma treatment are problematic for various reasons. First, it may take several months to uncover the response of a biologic agent in severe asthma patients who are at high risk of acute exacerbation during these periods. Second, it is impractical for patients of severe asthma with poor symptom control and high risk of acute exacerbation to undergo therapeutic trials because of the high costs of novel biologic agents.

Previous studies have confirmed the relative safety of performing bronchoscopy biopsies for patients with severe asthma [8]. Various sampling techniques have been used for tissue diagnosis, including nasal biopsies, nasal or bronchial brushing, and bronchoalveolar lavage [9]. Compared to these methods, bronchoscopy-guided bronchial epithelium sampling provides more accurate information about the epithelial and inflammatory cells in the tissue context. It is thus a powerful tool for selecting the most suitable targeted biologics in difficult clinical conditions.

## Supplementary information

**Additional file 1: Table S1.** Result of serial pulmonary function test for the patient. **Table S2.** Blood cell count and percentage for the patient.

**Additional file 2: Figure S1**. Small erythematous papules on trunk and extremities of the patient. **Figure S2.** Pathological finding for the skin rash. The biopsy specimen of an erythematous papules taken from right thigh showing (A) a superficial perivascular infiltrate  and (B) the infiltrate consisting of lymphocytes with eosinophils (A. H&E, X20; B. H&E, X200, zoom in from the black box in Fig. 2a).

## Data Availability

Data sharing is not applicable to this article as no datasets were generated or analysed during the current study.

## Abbreviations

ICS: inhaled corticosteroid
LAMA: long-acting muscarinic antagonist
LABA: long-acting beta-agonist
MAST: multiple-antigen simultaneous test
OCS: oral corticosteroid
PEF: peak expiratory flow
SABA: short-acting beta-agonist
