# Supplementary material for: Bronchoscopy-guided bronchial epithelium sampling as a tool for selecting the optimal biologic treatment in a patient with severe asthma: a case report
Source: Allergy Asthma Clin Immunol. 2019 Nov 27;15:76. doi: 10.1186/s13223-019-0378-6 (PMC6881985; doi:10.1186/s13223-019-0378-6)
Supplement: Supplementary file 1 — Additional file 1: Table S1. Result of serial pulmonary function test for the patient. Table S2. Blood cell count and percentage for the patient. [file 13223_2019_378_MOESM1_ESM.docx]

| Date of tests | Mar. 3, 2017 | Apr. 24, 2017 | Apr. 30, 2018 |
| --- | --- | --- | --- |
| Pre-bronchodilator test |  |  |  |
| FVC, L(%pred) | 2.03(84) | 3.15(130) | 2.30(89) |
| FEV_1_, L(%pred) | 0.91(47) | 1.82(93) | 0.96(43) |
| FEV_1_/FVC, % | 45 | 58 | 42 |
| Post-bronchodilator test |  |  |  |
| FVC, L(%chg) | 2.55(26) | 3.05(-3) | 2.39(4) |
| FEV_1_, L(%chg) | 0.94(2) | 1.72(-5) | 1.09(13) |
| FEV_1_/FVC, % | 37 | 56 | 46 |

**Table S1.** Result of serial pulmonary function test for the patient.

| Date | 2017-03-01 | 2017-04-07 | 2017-08-25 | 2017-09-19 | 2017-10-03 | 2017-12-25 | 2018-03-02 | 2018-05-07 | 2018-06-13 | 2018-07-09 | 2018-07-23 |
| --- | --- | --- | --- | --- | --- | --- | --- | --- | --- | --- | --- |
| WBC count (10^3^cell/μL) | 10.0 | 11.3 | 12.0 | 12.2 | 8.7 | 18.1 | 12.7 | 11.7 | 11.2 | 8.3 | 6.9 |
| Eos (%) | 4.1 | 0.0 | 6.8 | 1.8 | 3.3 | 0 | 1.1 | 14.2 | 1.7 | 3.7 | 7.5 |
| Eos count  (cell/μL) | 410 | 0^*^ | 816 | 219.6 | 287.1 | 0^**^ | 139.7 | 1661.4 | 190.4 | 307.1 | 517.5 |
| IgE level (IU/mL) | 42.6 | - | - | - | - | - | - | - | - | 160 | - |

FEV1: forced expiratory volume in one second, FVC: forced vital capacity

**Table S2.** Blood cell count and percentage for the patient.

Eos: eosinophil, IgE: immunoglobulin E, WBC: white blood cell

^*^ Because poor asthma control, the patient received daily oral methylprednisolone (8 mg) for 14 days and weekly intramuscular triamcinolone (40 mg) for 2 weeks before the blood eosinophil examination.

^**^ Because poor asthma control, the patient received twice-daily oral prednisolone (15 mg) for 7 days before the blood eosinophil examination.
