# Supplementary material for: Bronchoscopy-guided bronchial epithelium sampling as a tool for selecting the optimal biologic treatment in a patient with severe asthma: a case report
Source: Allergy Asthma Clin Immunol. 2019 Nov 27;15:76. doi: 10.1186/s13223-019-0378-6 (PMC6881985; doi:10.1186/s13223-019-0378-6)
Supplement: Supplementary file 2 — Additional file 2: Figure S1. Small erythematous papules on trunk and extremities of the patient. Figure S2. Pathological finding for the skin rash. The biopsy specimen of an erythematous papules taken from right thigh showing (A) a superficial perivascular infiltrate and (B) the infiltrate consisting of lymphocytes with eosinophils (A. H&E, X20; B. H&E, X200, zoom in from the black box in Fig. 2a). [file 13223_2019_378_MOESM2_ESM.pptx]

## Slide 1
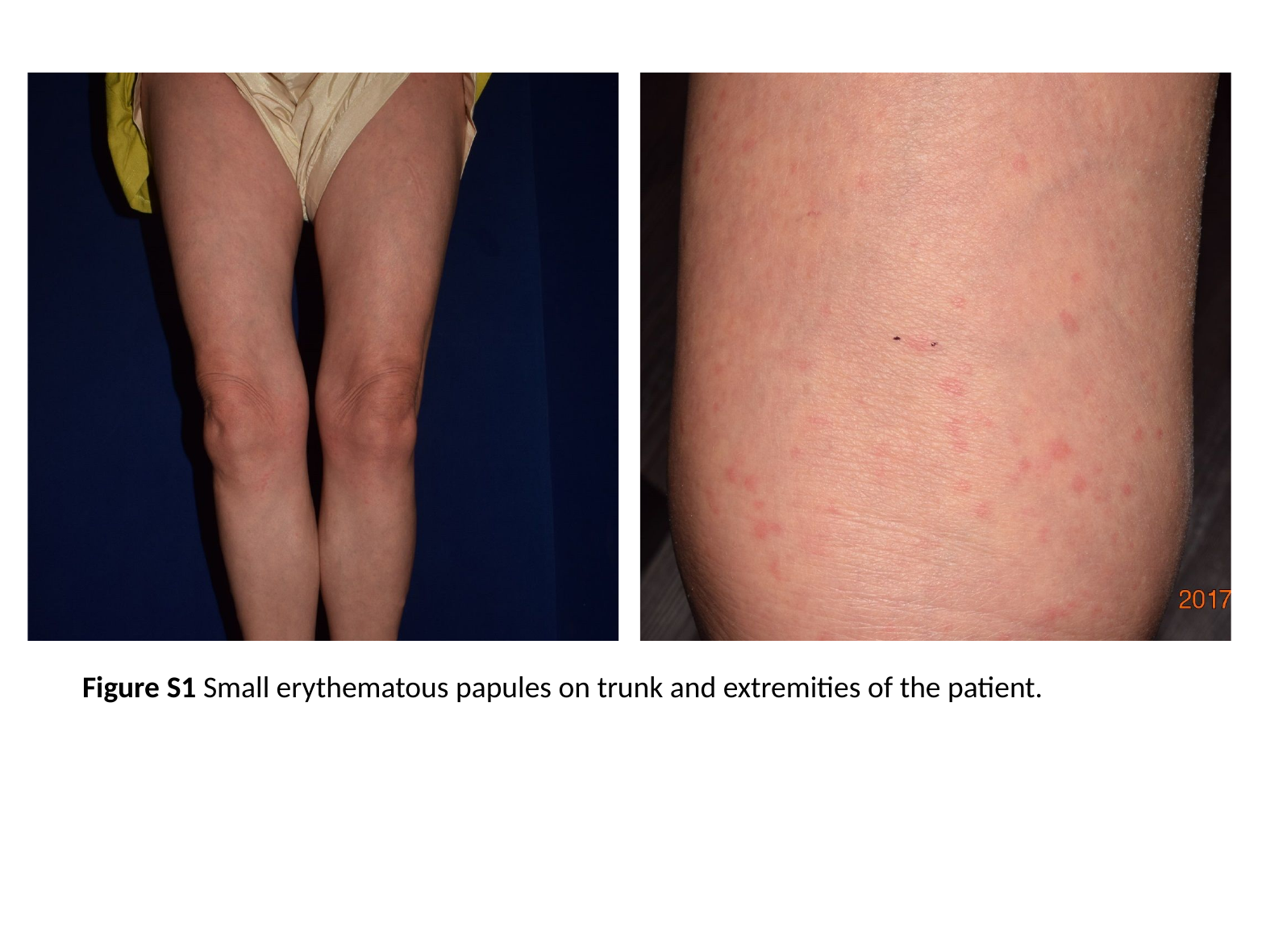

Figure S1 Small erythematous papules on trunk and extremities of the patient.

## Slide 2
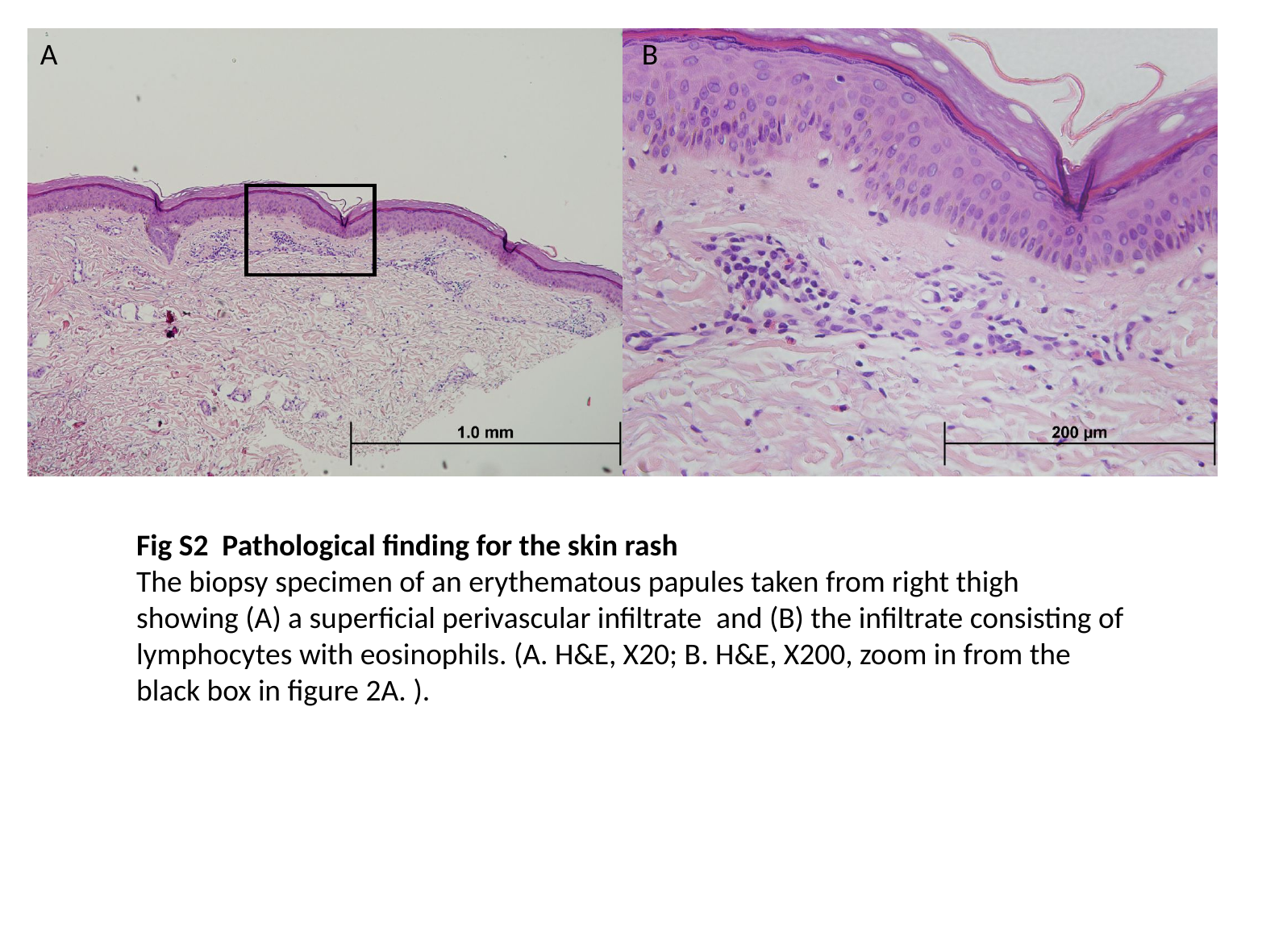

B
A
Fig S2 Pathological finding for the skin rash
The biopsy specimen of an erythematous papules taken from right thigh showing (A) a superficial perivascular infiltrate  and (B) the infiltrate consisting of lymphocytes with eosinophils. (A. H&E, X20; B. H&E, X200, zoom in from the black box in figure 2A. ).
